# Comparative analysis of lung function in supine and sitting positions in patients with moderate multiple sclerosis

**DOI:** 10.1177/20552173261443831

**Published:** 2026-04-16

**Authors:** Joar Wikars, Margareta Kånåhols, Ylva Nilsagård, Martin Gunnarsson, Elisabeth Westerdahl

**Affiliations:** School of Health Sciences, Faculty of Medicine and Health, Örebro University, Örebro, Sweden; Department of Physiotherapy, 59566 Örebro University Hospital, Örebro, Sweden; University Health Care Research Center, Faculty of Medicine and Health, Örebro University, Örebro, Sweden; Department of Neurology, Faculty of Medicine and Health, Örebro University, Örebro, Sweden; University Health Care Research Center, Faculty of Medicine and Health, Örebro University, Örebro, Sweden

**Keywords:** Body position, posture, pulmonary function tests, respiratory muscle strength, spirometry, vital capacity

## Abstract

**Background:**

Multiple sclerosis (MS) is a progressive disease that may affect respiratory muscle function. Pulmonary dysfunction may be subclinical, and posture changes can reduce lung volumes, especially in the presence of respiratory muscle weakness.

**Objective:**

To determine whether lung function differs between sitting and supine positions in individuals with moderate MS, and to explore associations with respiratory muscle strength and disability.

**Methods:**

Forty-eight participants with moderate MS (13 men, 35 women; median Expanded Disability Status Scale (EDSS) 4.5, range: 4.0–6.5) underwent spirometry (VC, FVC, FEV_1_, PEF) in sitting and supine positions. Respiratory muscle strength was assessed using maximal inspiratory (MIP) and expiratory pressures (MEP).

**Results:**

VC and FVC did not differ significantly between positions. FEV_1_ and PEF were slightly reduced in the supine position (*p* ≤ 0.001), with median relative decreases of −6% and −10%, respectively. These changes did not correlate with EDSS, MIP, or MEP. No sex-related differences were observed.

**Conclusion:**

FEV_1_ and PEF are reduced in the supine position in individuals with moderate MS, indicating early positional respiratory changes. These alterations appear independent of disability level or respiratory muscle strength. However, it remains uncertain whether these changes differ from those of healthy individuals. Controlled studies are warranted to clarify their clinical significance.

## Introduction

Multiple sclerosis (MS) can lead to respiratory muscle impairment through various mechanisms, including impaired motor control, fatigue, and physical inactivity.^1–3^ General muscle deconditioning may lead to impaired lung function, and a range of MS-related symptoms can further contribute to respiratory muscle dysfunction. Expiratory muscle weakness reduces cough effectiveness and, when combined with bulbar dysfunction, further compromises airway clearance. Cough efficacy has been shown to correlate with disability severity in MS.^4^ Although respiratory complications are most common in individuals with advanced MS, respiratory muscle weakness may be present at all stages of the disease.^5^ Respiratory disease has been identified as a leading cause of mortality in patients with MS.^6^

Respiratory impairment can be treated with chest physiotherapy, non-invasive ventilation, and preventive measures such as smoking cessation, nutrition, and respiratory muscle strength conditioning.^5,7,8^ However, systematic evaluation of respiratory function is not routinely implemented in clinical practice. Early identification of respiratory dysfunction may therefore be essential to optimize pulmonary function, prevent complications, and improve quality of life in individuals with MS.

Since respiratory function may be compromised in MS, early assessment is clinically important.^9^ Bedside spirometry using portable equipment provides a convenient method for this evaluation. Inspiratory capacity is typically measured via slow vital capacity (VC), while expiratory function is assessed with forced expiratory volume in one second (FEV_1_) and peak expiratory flow (PEF).^10^ Monitoring these parameters is crucial, even if changes in lung function are primarily seen in individuals with severe MS.^5,11^

In the supine position, VC may be reduced due to both mechanical and physiological changes affecting respiratory mechanics. Small but significant relative decreases in VC, FVC, FEV1, and PEF between the sitting and supine positions have been reported in healthy people.^12^

Respiratory muscle strength can vary with body position. In the supine position, abdominal contents may restrict diaphragmatic movement, potentially reducing maximal expiratory force and airflow, and thereby unmasking positional respiratory weakness.^13^ Additionally, increased pulmonary blood volume in the supine posture may reduce lung compliance through vascular congestion and decreased alveolar distensibility.^14^ These effects are particularly relevant in conditions such as MS, where respiratory muscle weakness may lead to subclinical pulmonary dysfunction even in the absence of overt symptoms.

Dysfunction of the diaphragmatic muscle may also limit the inspiratory ability and reduce the functional residual capacity (FRC).^15^ Further decrease in the supine position can potentially lead to airway closure and atelectasis, causing dyspnea.^16^ MS may lead to unilateral or bilateral dysfunction of the diaphragm hemispheres.^17,18^ A reduction in FVC of 15% between the supine and sitting positions is used as a diagnostic criterion for diaphragm dysfunction.^13^ If supine spirometry reveals a more pronounced decline in lung function, this may indicate a higher risk for conditions like nocturnal hypoventilation or sleep-disordered breathing.

Although respiratory impairment in MS is most pronounced in advanced stages, individuals with moderate disease may experience reduced respiratory muscle strength or positional lung function changes that are not detected by standard sitting spirometry.^5^ In this context, assessing lung function in both sitting and supine positions may reveal subtle deficits that are missed in routine evaluations.

This study aimed to determine whether lung function differs between sitting and supine positions in moderate MS, targeting subclinical respiratory dysfunction that may be missed in routine assessments, and to explore associations between these changes, respiratory muscle strength, and walking disability as assessed by the Expanded Disability Status Scale (EDSS).

## Materials and methods

This study employed a cross-sectional observational design with a within-subject comparison. The analysis was conducted using data previously collected as part of a research program investigating respiratory function in individuals with moderate MS. The previous research encompassed both an observational assessment of patients’ walking ability and respiratory function,^19,20^ as well as an interventional randomized trial of respiratory muscle training versus control.^21^ The current manuscript reports novel analyses of these respiratory measurements, including positional spirometry. As a secondary analysis of a previously conducted randomized controlled trial, no formal sample size calculation was performed, and the study is considered exploratory. The study was approved by the Regional Ethical Review Board in Uppsala (2012/077), which covered all respiratory assessments included in the present analysis, and written informed consent was obtained from all participants.

### Participants

The study cohort comprised 48 individuals with MS, aged ≥18 years and registered in the Swedish MS Registry, diagnosed according to the revised McDonald criteria^22^ and assessed as having moderate disease (EDSS 4.0–6.5). The inclusion criteria were three months without relapse, the ability to walk independently with or without walking aids, and the ability to understand written and spoken information. The exclusion criteria were additional neurological disease, orthopedic conditions, severe ischemic heart disease, and other cognitive difficulties which could impact the performance of lung function measurements. Patients were asked about any lung disease or smoking habits. EDSS scores were assessed by a neurologist.

### Procedures and measures

Slow and forced simple spirometry maneuvers were performed with a portable MicroLab spirometer (Micro Medical / CareFusion, Kent, UK) according to recommendations from the European Respiratory Society (ERS) and American Thoracic Society (ATS)^23^ in both the sitting and supine positions. The highest VC was taken from three correctly performed slow maneuvers. The highest FVC, FEV_1_, and PEF values were taken from three correctly performed forced maneuvers.

For the measurement of maximal inspiratory pressure (MIP) and maximal expiratory pressure (MEP), a micro respiratory pressure meter (Micro Medical/CareFusion) was used. Measurements were performed according to the ERS/ATS recommendations in a seated position.^24^ The highest MIP and MEP values were taken from five technically acceptable inspiratory maneuvers and five expiratory maneuvers, respectively.

### Statistical analysis

The collected data were assessed to be non-normally distributed. Data are presented in this article as median (Md) and interquartile range [IQR]. Wilcoxon signed-rank tests were performed to assess whether there was a significant difference in VC, FVC, FEV_1_, and PEF measurements between the seated and supine positions. Spearman's rank correlation was used to assess the association between EDSS and ΔVC, ΔFVC, ΔFEV_1_, and ΔPEF, as well as between each of the spirometric changes and MEP and MIP, respectively. Scatterplots were created for VC, FVC, FEV_1_, and PEF with their respective mean values for seated and supine positions on the *x*-axis and the corresponding relative difference between seated and supine on the *y*-axis. A Mann–Whitney *U*-test was performed to evaluate whether there was a significant difference in ΔVC, ΔFVC, ΔFEV_1_, and ΔPEF between men and women. All statistical calculations were performed in SPSS 28.0 for Windows (IBM Inc., Chicago, IL, USA).

## Results

The sample consisted of 13 men and 35 women with a median EDSS score of 4.5 (range: 4.0–6.5), representing moderate MS. Of these, 20 patients had relapsing-remitting MS, 26 had secondary progressive MS, and two had primary progressive MS. Four patients had an FEV_1_/VC ratio ≤70% of the expected value, indicating obstructive lung disease (Table 1).

**Table 1.** Characteristics of the study sample (*n* = 48).

Men/woman, *n*	13/35
Age, years	56 ± 11
BMI, kg/m^2^	27 ± 4
Never smoked/former smoker/current smoker, *n*	21/17/10
Pack-years	15 ± 11 [2–38]
Obstructive lung disease ^a^, *n*	*4
MS disease duration, years	24 ± 11
Relapsing-remitting MS, *n* (%)	20 (42%)
Secondary progressive MS, *n* (%)	26 (54%)
Primary progressive MS, *n* (%)	2 (4%)
EDSS, Md [IQR]	4.5 (4.0–6.5)
Relapse within three months, *n* (%)	2 (4%)

BMI: body mass index; EDSS: Expanded Disability Status Scale; IQR: interquartile range; Md: median; MS: multiple sclerosis.

a Obstructive lung disease was defined as FEV_1_/VC ≤ 70%.

There was no significant difference in VC or FVC between the seated and supine positions; however, FEV_1_ and PEF were significantly lower in the supine position compared to sitting (≤0.001). For FEV_1_, the relative difference between the seated and supine positions was −5.8% (IQR: −9.7% to −3.3%). The relative difference between the median PEF in the seated and supine positions was −9.5% (IQR: −14.7% to 0.5%; Table 2). Two patients with EDSS values of 4.0 and 7.5 had ΔVC ≥15%.

**Table 2.** Spirometry values for patients with moderate multiple sclerosis in sitting and supine position, respectively (*n* = 48).

	Sitting Md [IQR]	Supine Md [IQR]	Relative difference (Δ) between sitting and supine positions	*p*-value	*Z*-score
VC (L)	3.2 [2.73–3.86]	3.24 [2.80–3.64]	0.39% [−4.11% to 3.14%]	0.841	0.201
FVC (L)	3.26 [2.83–3.94]	3.27 [2.81–3.81]	−1.08% [−6.80% to 2.52%]	0.116	−1.572
FEV_1_ (L)	2.56 [2.12–3.05]	2.43 [1.99–2.77]	−5.83% [−9.68% to −3.32%]	<0.001	−5.181
PEF (L/Min)	364 [311–446]	348 [288–396]	−9.50% [−14.71% to 0.53%]	<0.001	−4.329

Data are presented as median (interquartile range). *p* < 0.05 was considered statistically significant. FEV_1_: forced expiratory volume in one second; FVC: forced vital capacity; IQR: interquartile range; L: liter; L/min: liters/minute; Md: median; MS: multiple sclerosis; PEF: peak expiratory flow; VC: vital capacity.

Spearman's rank correlation test did not show any significant correlations between EDSS values and ΔVC, ΔFVC, ΔFEV_1_, or ΔPEF. There were also no significant correlations between MEP or MIP and these variables (Table 3).

**Table 3.** Spearman's rank correlations between respiratory muscle strength (MEP, MIP), disability level (EDSS) and positional change in lung function from sitting to supine position in patients with moderate multiple sclerosis (*n* = 48).

	MEP (cmH_2_O)		MIP (cmH_2_O)		EDSS	
	*R*	*p*-value	*R*	*p*-value	*R*	*p*-value
ΔVC (L)	0.152	0.301	0.137	0.352	0.031	0.835
ΔFVC (L)	0.005	0.975	−0.042	0.776	0.054	0.715
ΔFEV_1_ (L)	0.011	0.939	0.049	0.741	−0.144	0.327
ΔPEF (L/min)	−0.039	0.792	−0.125	0.397	−0.274	0.060

Data are presented as differences (Δ) between sitting and supine positions. *p* < 0.05 was considered statistically significant. EDSS: Expanded Disability Status Scale; FEV_1_: forced expiratory volume in one second; FVC: forced vital capacity; MEP: maximal expiratory pressure; MIP: maximal inspiratory pressure; *r*: coefficient of correlation; PEF: peak expiratory flow; *R* = Spearman's rank correlation coefficient (ρ); VC: vital capacity.

**Figure 1.**
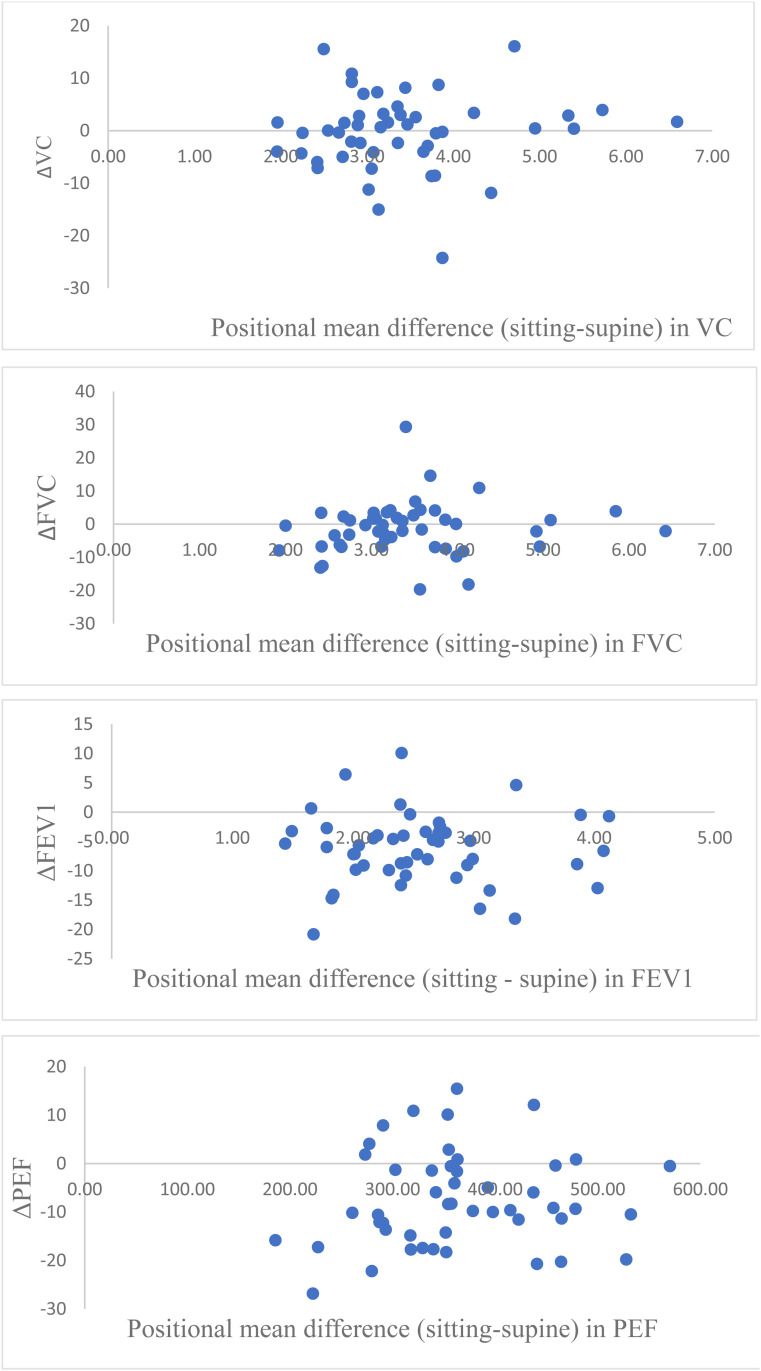
Scatter plots showing changes in lung function between sitting and supine positions in patients with multiple sclerosis (*n* = 48). Each panel presents the change (Δ): ΔVC: vital capacity, ΔFVC: forced vital capacity, ΔFEV_1_: forced expiratory volume in one second, and ΔPEF: peak expiratory flow. The *x*-axis shows the mean value of the relevant variable across both positions for each participant, and the *y*-axis represents the positional difference (sitting minus supine), where positive values indicate higher values in the sitting position.

The Mann–Whitney *U*-test showed no significant difference in ΔVC, ΔFVC, ΔFEV_1_, or ΔPEF between men and women (*p* = 0.935). Visual assessment of scatterplots showed no covariation between the size of lung function values and the relative difference in lung function values between sitting and supine positions (Figure 1).

## Discussion

This study demonstrated slight differences in PEF and FEV_1_ between sitting and supine positions in individuals with moderate MS. Although previous research has assessed respiratory parameters across a wider EDSS range, data specifically examining the impact of postural changes in this population remain limited. In contrast, VC and FVC did not differ significantly between sitting and supine positions. The reductions in PEF and FEV_1_ in the supine position may reflect posture-related alterations in respiratory mechanics, including decreased diaphragmatic excursion and reduced contribution of the abdominal muscles, which may be exacerbated by MS-related neuromuscular weakness.

A study conducted on 84 MS patients with suspected diaphragm dysfunction found that ΔVC had 87% sensitivity but only 52% specificity for detecting unilateral diaphragm dysfunction, while the sensitivity and specificity for bilateral diaphragm dysfunction were 100% and 89%, respectively.^25^ Additionally, people with MS have been reported to exhibit a slightly greater ΔVC compared to healthy controls.^26^ Another study found a significant correlation between ΔFVC and the degree of MS-related disability as measured by the EDSS.^27^ These findings underscore the importance of considering posture when assessing respiratory function in MS, and highlight potential implications for airway clearance strategies and respiratory rehabilitation.

Hashim et al.^27^ reported a significant difference between sitting and supine positions in individuals with mild to moderate MS, with a mean change of −15% ± 6%. Levy et al.,^11^ however, found no significant decrease in VC between these positions in severely affected patients who used a wheelchair full-time. Maillart et al.^17^ observed that patients with severe MS and diaphragmatic dysfunction experienced a significant relative decrease in VC while sitting compared to supine. Our findings differ from some previous studies in that VC and FVC were not significantly affected by posture, while FEV1 and PEF decreased in the supine position. This may reflect differences in population characteristics, and in measurement sensitivity, with flow-dependent variables being more responsive to positional changes than volume-based measures. These factors may help explain the discrepancies with prior studies and highlight the importance of examining positional effects specifically in the moderate MS population.

Although previous studies on postural effects in MS show inconsistent results, our study specifically targets individuals with moderate MS. Our findings suggest that flow-dependent measures are particularly sensitive to posture, providing novel data that help reconcile discrepancies in the literature.

Lung function in healthy individuals has been shown to differ between postural positions. Most of the studies included in a systematic literature review by Katz et al.^12^ found a significant difference in ΔPEF and ΔFEV_1_ between sitting and supine positions in healthy individuals. Since our study lacked a healthy control group, it is not possible to determine whether the observed postural decreases in lung function are specific to MS or reflect normal physiological variation. However, Altintas et al.^26^ found a significant difference in ΔVC between individuals with mild MS and healthy controls.

Maillart et al.^17^ found that of 71 patients with severe MS, about half had some degree of diaphragmatic dysfunction. Four patients had a VC/FEV_1_ ratio indicating obstructive lung disease according to the GOLD criteria.^28^ Melam et al.^29^ found that asthma patients had lower FEV_1_ and FVC values in the supine position compared to sitting. We are not aware of any studies comparing positional lung function changes in obstructive patients versus healthy controls, so we cannot determine whether obstructiveness influenced our results.

No correlations could be found between EDSS, MIP, or MEP and ΔFVC, ΔVC, ΔFEV_1_, or ΔPEF. This is in contrast to the results of Hashim et al.^27^ who found a significant correlation between EDSS and ΔFVC in people mildly to moderately affected by MS (but did not evaluate ΔVC, ΔFEV1, or ΔPEF). The larger sample size in Hashim et al.'s study (n = 149) likely contributed to the detection of significant associations.

## Limitations

Several limitations should be considered. The study included a relatively small sample restricted to a narrow EDSS range, which may limit generalizability to individuals with milder or more advanced MS. The absence of a healthy control group prevents distinguishing MS-specific postural effects from normal physiological variation. The cross-sectional design does not allow assessment of longitudinal changes or causal relationships. Although standard volume- and flow-dependent measures were assessed, additional parameters such as expiratory reserve volume (ERV) and FRC were not measured and could provide further insight into positional effects. Finally, the study may have been underpowered to detect correlations between EDSS, MIP, or MEP and postural changes in respiratory function.

Despite these limitations, the study provides novel evidence that flow-dependent measures are particularly sensitive to posture in moderate MS, highlighting the value of assessing respiratory function in multiple positions and informing future research and rehabilitation strategies. The results are mainly applicable to individuals with moderate MS and should be interpreted cautiously when extrapolating to patients with more advanced disease, who have been shown to exhibit more pronounced impairments in lung function.^11,17^

Current pulmonary assessments in MS often rely on sitting spirometry, which may not fully capture the functional respiratory status of some individuals. Comparing values in both positions may improve the sensitivity of pulmonary function tests in detecting MS-related respiratory decline. Different body positions affect lung volumes, airflow, respiratory mechanics, abdominal pressure, and expiratory muscle activation, and therefore, the flow-dependent measures appear more responsive to changes in airway mechanics or diaphragmatic function in the supine posture, whereas volume-based measures remain relatively stable. Further studies are needed to clarify the mechanisms underlying these positional effects.

Although respiratory muscle training has been shown to enhance respiratory muscle strength in individuals with MS, and lung volume recruitment may help slow the decline in vital capacity, there remains a lack of evidence to guide the development of respiratory rehabilitation programs tailored to varying levels of disability associated with the disease.^7^

Future studies could explore ERV and FRC in both sitting and supine positions, as these volumes typically decrease when supine, sometimes causing small airway closure, and people with MS may show larger differences between postures. Such data could inform the design of more practical and effective preventive breathing interventions for this population.

## Conclusion

In patients with moderate MS, lung function as measured by FEV_1_ and PEF is significantly reduced in the supine position, suggesting early positional respiratory changes despite overall preserved lung volumes. These changes do not appear to correlate with EDSS or respiratory muscle strength, indicating that positional spirometry may detect subtle respiratory involvement. Exploratory analyses of positional respiratory changes by type of disability could be of interest in future studies, and controlled studies are warranted to further clarify these findings.
